# Factors Associated with Depressive Symptoms Among Korean Young Adults by Sex in the Third Year of the COVID-19 Pandemic

**DOI:** 10.3390/healthcare14193356

**Published:** 2026-10-08

**Authors:** Seol-Bin Kim

**Affiliations:** College of Nursing, Geoje University, Geoje 53325, Republic of Korea; ksb0061@naver.com

**Keywords:** depression, young adult, sex factors, COVID-19, Republic of Korea

## Abstract

**Background/Objectives:** This study analyzed factors associated with depressive symptoms by sex among Korean young adults in the third year of the coronavirus disease 2019 (COVID-19) pandemic. **Methods:** The subjects of this secondary data analysis were 47,353 young adults (23,407 men and 23,946 women) aged 19–39 years from the nationwide 2022 Community Health Survey. Depressive symptoms were defined as a Patient Health Questionnaire-9 score ≥ 6. Complex sample multiple logistic regression analyses were performed for the total sample (adjusted for sex) and separately for men and women. **Results:** The proportion of participants with depressive symptoms was 16.3% in women and 10.1% in men, and women had higher odds of depressive symptoms than men (adjusted odds ratio = 1.89, 95% confidence interval: 1.72–2.07). In both sexes, high subjective stress, poor subjective health status, short or long sleep duration, perceiving oneself as obese, living alone, low household income, and smoking were associated with depressive symptoms, whereas a higher body mass index (obesity in men and overweight in women) was associated with lower odds. Lower education; being separated, widowed, or divorced; having no occupation; and perceiving oneself as skinny were associated with depressive symptoms only in men, whereas binge drinking was associated with depressive symptoms only in women. **Conclusions:** Korean young adults showed both common and sex-specific correlates of depressive symptoms in 2022. Screening and intervention programs for depressive symptoms in young adults may need to consider psychological stress, sleep, and economic and household conditions, as well as sex differences.

## 1. Introduction

### 1.1. Background

Depressive disorder is a common mental health disorder encompassing depressive moods and a loss of interest in activities and affecting all aspects of one’s life [1]. As of 2019, approximately 280 million people worldwide were estimated to be suffering from depressive disorder, and depressive disorder accounted for the highest portion of the disease burden caused by mental health disorders [2]. Globally, the number of cases of depressive disorder increased by approximately 50% between 1990 and 2017, and South Korea showed the third-highest increase in the age-standardized incidence of depressive disorder during this period [3]. According to the South Korean Mental Health Survey [4], the lifetime prevalence of depressive disorder increased from 5% in 2016 to 7.7% in 2021, an increase of 2.7 percentage points. Furthermore, in 2019, the number of patients aged 15–54 years old suffering from depressive disorder in South Korea crossed 500,000, and the social loss until these patients turned 55 years old was estimated to be 133 trillion KRW [5], indicating depressive disorder’s considerable socioeconomic impact.

The COVID-19 pandemic was associated with a significant increase in the global prevalence and burden of depressive disorder, particularly among younger age groups [6]. In South Korea, depressive moods among young adults increased significantly in 2021, the second year of the pandemic, compared with 2019, before the pandemic [7]. According to Erikson’s theory of psychosocial development, young adulthood is the stage of intimacy versus isolation and an important period for forming social relationships [8]. However, social distancing policies during the pandemic [9] restricted social activities in many areas, which may have had implications for the psychosocial development of young adults. According to the 2022 National Mental Health Knowledge and Attitudes Survey [10], young adults in their 20s and 30s reported the highest rate of mental health problems among all age groups, and those who experienced such problems reported that the areas most impeded were “work and studies” among those in their 20s and “social activities and interpersonal relationships” among those in their 30s. These findings suggest that the mental health of young adults remained a concern in 2022, the third year of the pandemic.

For young adults suffering from depressive disorder, psychosocial intervention significantly reduces depressive symptoms [11], and psychotherapy by itself can have a therapeutic effect on mild depressive symptoms, rendering the prescription of antidepressants unnecessary [1]; this indicates that early treatment is important. However, among South Koreans diagnosed with depressive disorder, the ratio of individuals using mental health services is only 12.5% [12]; that is, most are not receiving suitable interventions. In a prospective study of a community cohort [13], most adolescents and young adults with mental health problems (77.5%) displayed help-seeking behaviors: They requested mental health services only after experiencing the worsening of psychiatric symptoms and developing functional impairment. This suggests that the screening of individuals at high risk for depressive disorder or patients with early depressive disorder for referral to mental health services is an effective strategy. Moreover, once they become more aware of the difficulties posed by mental health issues, young women are more likely to use mental health services, whereas young men are less likely to do so [14]. Therefore, efforts to detect and prevent depressive disorder among young adults should account for sex differences.

General factors associated with depressive disorder include age, education, marital status, occupation, household income, and residential environment [15,16,17,18]. South Korean studies examining the relationship between health behaviors and depressive disorder associate smoking, physical activity, and body mass index (BMI) with depressive disorder in both men and women [15] and indicate that high-risk drinking is associated with a 1.52-fold higher risk of depressive disorder [16]. Further, a meta-analysis of the relationship between sleep duration and mental health disorders associated short sleep duration with a higher risk of depressive disorder compared with normal sleep duration [19]. Among psychosocial characteristics, subjective health status and perceived stress were associated with depressive disorder [15]. Regarding subjective body image, men idealized a larger body shape, whereas women preferred a slimmer body shape, and dissatisfaction with body shape was associated with depressive disorder in both groups [20]. In particular, the COVID-19 pandemic widened the sex gap in various areas, with respect to physical activity, health-related behaviors, mental health, and health communication [21]. Internationally, the COVID-19 pandemic was associated with larger increases in the prevalence of depressive disorders among women and younger age groups [6], and a longitudinal population survey in the United Kingdom found that mental distress increased most among adults aged 18–34 years and among women [22]. However, a systematic review of studies conducted in the early phase of the pandemic reported that changes in depressive symptoms did not differ, or were worse only by small amounts, among women compared with men [23], indicating that the evidence on sex differences in depression during the pandemic remains inconsistent.

Although several South Korean studies have examined the factors associated with depression using Community Health Survey data [15,16], few have examined these factors among young adults separately by sex since the onset of the COVID-19 pandemic. An earlier study of young adults aged 19–39 years [16] used pre-pandemic data from the 2017 Community Health Survey (Table S1), and a nationwide study of health behaviors and depression [15] included adults of all ages. Given that depressive moods among young adults increased significantly in 2021 compared with the pre-pandemic period [7], continued monitoring using more recent data is warranted. Moreover, because young men and women differ in their use of mental health services [14], the COVID-19 pandemic widened sex gaps in mental health and health-related behaviors [21], and some factors associated with depression differ by sex [15,20], identifying the factors common to both sexes and those specific to each sex may help tailor screening and intervention strategies. Therefore, this study used data from the 2022 Community Health Survey to examine the factors associated with depressive symptoms among Korean young adults, first in the total sample and then separately for men and women, to provide basic data for depressive symptom screening and intervention in this population.

### 1.2. Research Objectives

This study used data from the 2022 Community Health Survey to identify common and sex-specific factors associated with depressive symptoms among young adults in the third year of the COVID-19 pandemic. The following specific research objectives were examined:

- To ascertain participants’ general characteristics, health-related characteristics, psychological characteristics, and depressive symptoms by sex.
- To identify the differences in depressive symptoms by sex depending on the participants’ general, health-related, and psychological characteristics.
- To identify the factors associated with the participants’ depressive symptoms in the total sample and by sex.

## 2. Materials and Methods

### 2.1. Study Design

This cross-sectional study used secondary data from the 2022 Community Health Survey to identify the factors associated with depressive symptoms among young adults by sex in the third year of the COVID-19 pandemic.

### 2.2. Study Population and Sampling

**Figure 1:**
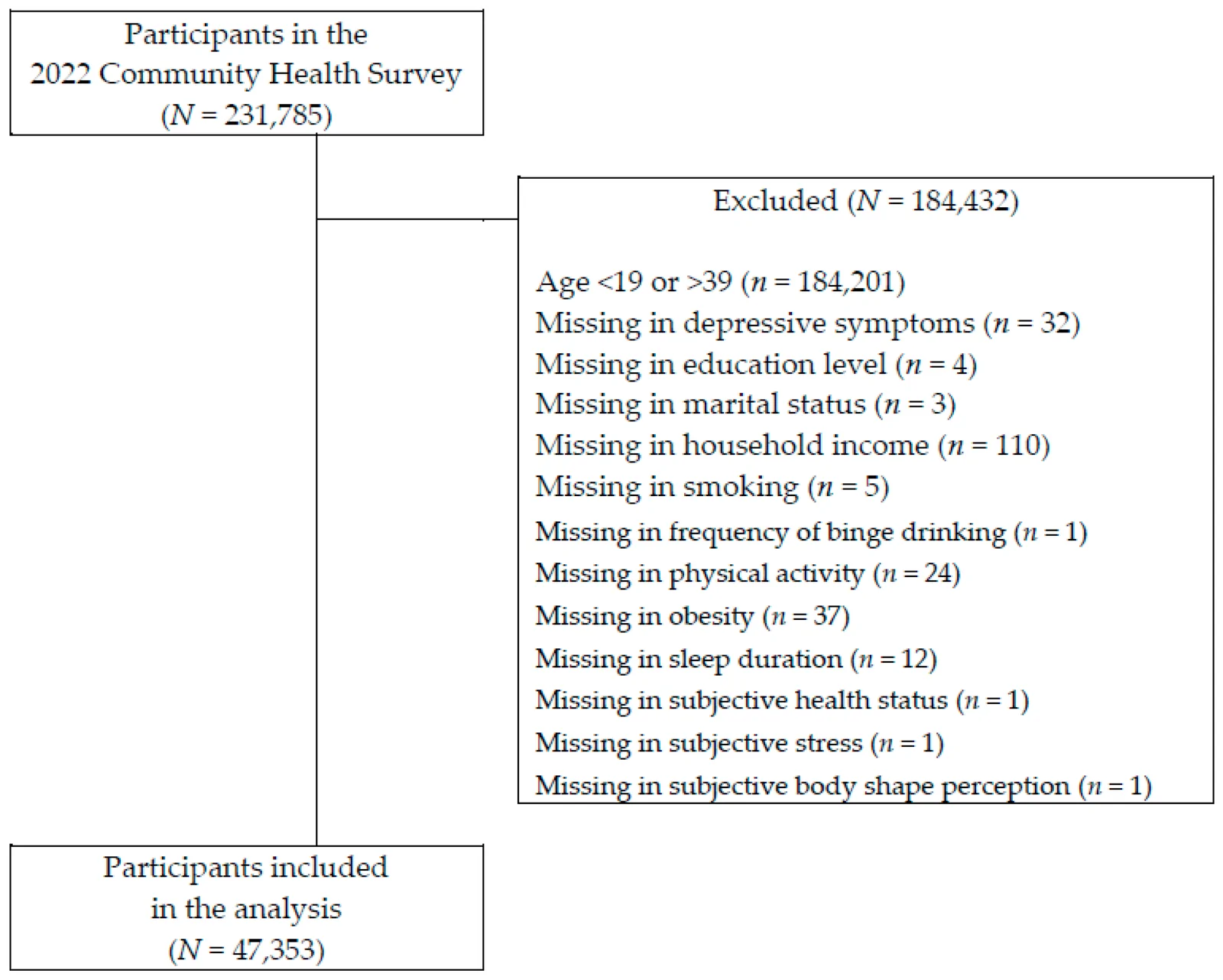
The Flowchart of Participant Selection.

We used raw data from the 2022 Community Health Survey. The primary data source Community Health Survey is conducted on a national sample in city/county/district units to ascertain residents’ health status and produce the health statistics data required to establish and evaluate evidence-based healthcare policies. The survey has been conducted annually since 2008. This survey’s target population was adults aged 19 years or older living in residential housing in South Korean territory. Resident registration-based population data from the Ministry of the Interior and Safety and housing data from the Ministry of Land, Infrastructure and Transport were linked to obtain a stratified (dong/eup/myeon, residence type), clustered (household) sample. All members of the selected households were included as survey participants. The survey content included both individual and household surveys. The individual survey consisted of a questionnaire, anthropometric measurements, and blood pressure measurement. The data collection period for the 2022 Community Health Survey was from 16 August to 31 October 2022. Trained survey supervisors directly visited the selected households and performed face-to-face computer-assisted personal interviewing. The survey was completed by a total of 231,785 participants. Based on the age criterion of 19–39 years for young adults used in an earlier study in South Korea [16], 47,584 participants aged 19–39 years were identified. After excluding 231 participants (0.5%) with missing values for any of the study variables (complete-case analysis), 47,353 participants (23,407 men and 23,946 women) were included in the final analysis. The participant selection process is shown in Figure 1.

### 2.3. Research Instruments

#### 2.3.1. General Characteristics

Age, education, marital status, occupation, household income, size of household, and area of residence were included as general participant characteristics. Age was categorized as either “19–29 years” or “30–39 years”; education was “below university graduation” or “university graduation or higher”; marital status was “single,” “married,” or “separated, widowed, or divorced”; and occupation was “yes” or “no” depending on whether the participant was engaged in economic activities. Household income was assessed as the average monthly household income in the previous year; responses provided as either an exact amount or an income category were combined into a single variable. In 2022, the average monthly household income in South Korea was 4.8 million KRW [24]; hence, the data were recategorized as “<5 million KRW” or “≥5 million KRW.” Further, household size was categorized as either “1 person” or “≥2 persons.” Additionally, the areas of residence were categorized as “metropolitan” for participants living in a region designated as a Special City or Metropolitan City, “middle and small” for those living outside these regions in a dong administrative unit, and “rural” for those living in a eup or myeon administrative unit.

#### 2.3.2. Health-Related Characteristics

Smoking status, binge drinking, physical activity, obesity, and sleep duration were included as health behaviors. Among them, smoking status was recategorized as “nonsmoker” for participants with earlier or no smoking experience and “smoker” for current smokers. Binge drinking was defined as the frequency of consuming seven or more drinks (men) or five or more drinks (women) on a single occasion, and it was categorized into “never,” “up to once per week,” or “almost every day.” Physical activity data was reported in terms of metabolic equivalent task (MET), which was converted from the number of days and daily time spent on vigorous physical activity, moderate physical activity, and walking during the past week. Subsequently, MET was categorized in accordance with the scoring system of the International Physical Activity Questionnaire [25] as “inactive (MET < 600),” “minimally active (600 ≤ MET < 3000),” or “healthy (MET ≥ 3000).” Obesity was assessed using body mass index (BMI), calculated as weight in kilograms divided by the square of height in meters (kg/m^2^). In accordance with the 2022 clinical practice guidelines of the Korean Society for the Study of Obesity [26], obesity was categorized as “underweight (<18.5 kg/m^2^)”, “normal (18.5–22.9 kg/m^2^)”, “overweight (23.0–24.9 kg/m^2^)”, or “obese (≥25.0 kg/m^2^).” Finally, sleep duration was categorized as “<7 h,” “7–9 h” or “>9 h” based on the recommended daily sleep duration for adults of 7–9 h [27].

#### 2.3.3. Psychological Characteristics

In this study, psychological characteristics included subjective health status, subjective stress, and subjective body shape perception. The question “What do you think about your everyday health?” examined subjective health status, and participants’ responses were “Very good,” “Good,” “Average,” “Bad,” or “Very bad.” During data analysis, the participants who responded “Very good” or “Good” were categorized as “Good,” those who responded “Average” were categorized as “Average,” and the ones who responded “Very bad” or “Bad” were “Bad.” Further, to evaluate subjective stress, the participants were asked to mention the level of stress usually experienced by them in daily life. Their responses were “Very much,” “Much,” “A little,” or “Almost none.” Data analysis indicated that the participants who responded “Very much” or “Much” were categorized as having “High” subjective stress, and those who responded “A little” or “Almost none” had “Low” subjective stress. Similarly, subjective body shape perception was investigated by posing the question “How do you currently feel about your body shape?” The participants responded with “Very skinny,” “Somewhat skinny,” “Normal,” “Somewhat obese,” or “Very obese.” Following data analysis, the participants who responded “Very skinny” or “Somewhat skinny” were categorized as “Skinny,” those who responded “Normal” were categorized as “Normal,” and those who responded “Somewhat obese” or “Very obese” were categorized as “Obese.”

#### 2.3.4. Depressive Symptoms

The outcome variable of depressive symptoms was assessed using the total score for the Patient Health Questionnaire-9 (PHQ-9) [28]. Kim et al. [29] noted that optimal PHQ-9 cutoff scores tend to be lower in East Asian populations, including Korea, and suggested that this may be related to cultural factors, such as the stigma attached to mental health problems. Upon applying a cutoff score of 6 points, PHQ-9 shows a sensitivity of 91.5% in South Korean adults [30]. Since high sensitivity is effective in depressive symptoms screening [30], we categorized participants based on a cut-off score of 6 points into “No depressive symptoms (<6 points)” or “Depressive symptoms (≥6 points).”

### 2.4. Data Collection and Ethical Considerations

The guidelines of the Community Health Survey mandated the collection of written informed consent from all participants prior to data collection, and the collected raw data were anonymized such that they did not include any identifying information. This study was exempted from review by the Institutional Review Board (1041495-202406-HR-01-01) because it used anonymized secondary data. We obtained the approval of the Korea Disease Control and Prevention Agency (KDCA) to download the data for analysis from the KDCA homepage.

### 2.5. Data Analysis

IBM SPSS Statistics 26.0 (IBM Corp., Armonk, NY, USA) was used for data analysis, and statistical tests were performed at a two-tailed significance level of 0.05. The Community Health Survey data used in this study have a complex sample design; hence, they were analyzed based on complex sampling using weights, strata, and clusters:

Participants’ general characteristics, health-related characteristics, psychological characteristics, and depressive symptoms were analyzed by sex using complex sample descriptive statistics.

To investigate differences in depressive symptoms depending on general, health-related, and psychological characteristics by sex, we obtained unweighted frequencies and weighted percentages and used the Rao–Scott chi-square test, which can provide stable statistics for complex sample designs [31].

To identify the factors associated with depressive symptoms, multiple logistic regression analyses were performed with depressive symptoms as the outcome variable. Candidate covariates were selected a priori on the basis of previous studies reporting their associations with depression, including sociodemographic characteristics, health behaviors, and psychological characteristics [15,16,17,18,19,20]. Among these, variables that showed significant differences in the bivariate analysis for men or women were entered as independent variables. First, a pooled model including all participants was fitted with sex as an additional covariate. Second, sex-stratified models were fitted separately for men and women using the same set of covariates, excluding sex. Adjusted odds ratios (aORs) and 95% confidence intervals (CIs) were calculated.

## 3. Results

### 3.1. Participants’ General Characteristics, Health-Related Characteristics, Psychological Characteristics, and Depressive Symptoms

Table 1 presents the characteristics of the 47,353 participants (23,407 men and 23,946 women). The age distribution did not differ by sex (χ^2^ = 1.56, *p* = 0.212), whereas all other general characteristics differed significantly between men and women (all *p* < 0.001). Compared with women, men were more likely to have an education level below university graduation (59.1% vs. 52.3%), to be single (70.8% vs. 59.2%), and to live in a one-person household (18.8% vs. 12.5%).

Sex differences were most pronounced in health-related characteristics, all of which differed significantly by sex (all *p* < 0.001). The prevalence of smoking was approximately five times higher in men than in women (38.6% vs. 7.5%), and men were more likely to engage in binge drinking (up to once per week or almost every day: 68.5% vs. 54.2%) and to be physically active at a healthy level (30.8% vs. 14.3%). According to the Asian BMI criteria, 45.0% of men and 17.5% of women were obese (BMI ≥ 25.0 kg/m^2^), whereas underweight was more common in women than in men (12.7% vs. 2.1%). Short sleep duration (<7 h) was slightly more common in men than in women (40.3% vs. 35.4%).

Women reported less favorable psychological characteristics than men: fewer women rated their health as good (59.1% vs. 65.1%), and more women reported high subjective stress (29.4% vs. 24.4%) (both *p* < 0.001). Notably, although far fewer women than men were obese based on BMI (17.5% vs. 45.0%), the proportion of women who perceived their body shape as obese (39.2%) was approached that of men (45.8%), indicating a larger gap between measured and perceived body shape among women. The proportion of participants with depressive symptoms (PHQ-9 ≥ 6) was 13.0% overall and was significantly higher in women than in men (16.3% vs. 10.1%; χ^2^ = 312.06, *p* < 0.001). Overall, young men and women differed considerably in health-related and psychological characteristics as well as in the proportion of participants with depressive symptoms, which supports examining the factors associated with depressive symptoms separately by sex (Table 1).

### 3.2. Differences in Depressive Symptoms Based on Participants’ General, Health-Related, and Psychological Characteristics

In the bivariate analyses, depressive symptoms were associated with most general, health-related, and psychological characteristics in both men and women (Table 2). The largest differences were observed for psychological characteristics. Approximately two-thirds of participants with depressive symptoms reported high subjective stress (men: 66.1%; women: 67.4%), compared with approximately one-fifth of those without depressive symptoms (19.7% and 22.1%, respectively; both *p* < 0.001). Likewise, the proportion reporting bad subjective health status was more than five times higher among participants with depressive symptoms (men: 15.9% vs. 3.0%; women: 16.1% vs. 2.5%). In both sexes, participants with depressive symptoms were also more likely to sleep less than 7 h (men: 51.0% vs. 39.2%; women: 48.5% vs. 32.9%), to have a household income < 5 million KRW (men: 57.8% vs. 49.4%; women: 56.6% vs. 45.2%), and to live in a one-person household (men: 23.8% vs. 18.2%; women: 18.4% vs. 11.4%).

Some associations differed by sex. The association with smoking appeared stronger in women: the proportion of smokers among women with depressive symptoms was nearly three times that among women without depressive symptoms (16.6% vs. 5.8%), whereas the corresponding difference was smaller in men (49.9% vs. 37.3%). Occupation was associated with depressive symptoms only in women (χ^2^ = 4.02, *p* = 0.045; men: χ^2^ = 1.40, *p* = 0.237), and the area of residence was not associated with depressive symptoms in either sex (men: *p* = 0.589; women: *p* = 0.065). Obesity based on BMI was more common among participants with depressive symptoms in both sexes (men: 48.8% vs. 44.4%; women: 24.3% vs. 16.2%). These results suggest that, whereas psychological characteristics were consistently associated with depressive symptoms regardless of sex, the relevance of some socioeconomic and behavioral characteristics differed between men and women.

### 3.3. Factors Associated with Participants’ Depressive Symptoms

Table 3 presents the results of the multiple logistic regression analyses. In the pooled model including all participants and adjusted for sex and all other variables, women had higher odds of depressive symptoms than men (adjusted OR [aOR] = 1.89, 95% CI: 1.72–2.07). Bad subjective health status (aOR = 6.20, 95% CI: 5.49–7.00) and high subjective stress (aOR = 5.61, 95% CI: 5.23–6.02) showed the strongest associations with depressive symptoms, followed by sleep duration > 9 h (aOR = 1.91) or <7 h (aOR = 1.49), perceiving one’s body shape as obese (aOR = 1.61), being separated, widowed, or divorced (aOR = 1.44), binge drinking almost every day (aOR = 1.41), smoking (aOR = 1.39), living in a one-person household (aOR = 1.25), and a household income < 5 million KRW (aOR = 1.22). In contrast, obesity based on BMI was associated with lower odds of depressive symptoms (aOR = 0.80, 95% CI: 0.71–0.90).

In the sex-stratified models including the same covariates, seven factors were associated with depressive symptoms in both men and women in the same direction: high subjective stress (men: aOR = 6.16, 95% CI: 5.52–6.88; women: aOR = 5.28, 95% CI: 4.82–5.79), average or bad subjective health status (men: aOR = 2.68 and 5.65; women: aOR = 2.33 and 6.61), sleep duration < 7 h or >9 h (men: aOR = 1.39 and 1.93; women: aOR = 1.57 and 1.89), perceiving one’s body shape as obese (men: aOR = 1.63; women: aOR = 1.65), living in a one-person household (men: aOR = 1.23; women: aOR = 1.27), a household income < 5 million KRW (men: aOR = 1.17; women: aOR = 1.24), and smoking. The association with smoking was notably stronger in women (aOR = 1.92, 95% CI: 1.67–2.22) than in men (aOR = 1.16, 95% CI: 1.04–1.29).

Other factors were associated with depressive symptoms in only one sex. Among men, an education level below university graduation (aOR = 1.27, 95% CI: 1.13–1.42), being separated, widowed, or divorced (aOR = 2.42, 95% CI: 1.71–3.42), having no occupation (aOR = 1.16, 95% CI: 1.01–1.33), and perceiving one’s body shape as skinny (aOR = 1.43, 95% CI: 1.19–1.72) were associated with higher odds of depressive symptoms, whereas obesity based on BMI was associated with lower odds (aOR = 0.76, 95% CI: 0.62–0.92). Among women, binge drinking up to once per week (aOR = 1.19, 95% CI: 1.08–1.31) or almost every day (aOR = 1.60, 95% CI: 1.23–2.08) was associated with higher odds, and being overweight was associated with lower odds of depressive symptoms (aOR = 0.82, 95% CI: 0.70–0.97). Age and physical activity were not associated with depressive symptoms in either sex. Taken together, psychological factors, sleep duration, smoking, and economic and household conditions were common correlates of depressive symptoms in young adults of both sexes, whereas education, marital status, and occupation were relevant only in men, and binge drinking only in women.

## 4. Discussion

This study used data from the 2022 Community Health Survey to identify the factors associated with depressive symptoms among Korean young adults by sex in the third year of the COVID-19 pandemic. Women had higher odds of depressive symptoms than men, and psychological characteristics, sleep duration, smoking, and economic and household conditions were common correlates of depressive symptoms in both sexes, whereas some socioeconomic and behavioral factors were associated with depressive symptoms in only one sex. These findings are discussed below in relation to previous studies.

In this study, 13.0% of young adults (men: 10.1%; women: 16.3%) screened positive for depressive symptoms (PHQ-9 score ≥ 6). Because depressive symptoms were defined using a screening cutoff that prioritizes sensitivity, this figure represents the proportion of young adults with a positive screening result rather than the prevalence of depressive disorder, and it should not be directly compared with estimates based on diagnostic interviews or different cutoff points. Nevertheless, the higher proportion and higher adjusted odds of depressive symptoms among women than among men are consistent with international evidence that women experienced greater increases in depressive disorders and mental distress during the COVID-19 pandemic [6,22].

Among the general characteristics, a household income below the national average (<5 million KRW/month) and living in a one-person household were associated with higher odds of depressive symptoms in both men and women. This is consistent with earlier studies reporting that economic burden is associated with depression [18]. In particular, during the COVID-19 pandemic, the prevalence of depression was at least four times higher among persons with few assets and low income than among those with more assets and higher income [32]. Therefore, our finding is consistent with reports linking financial hardship during the COVID-19 pandemic to poorer mental health outcomes [32].

The association with living in a one-person household differs from that of an earlier pre-pandemic study on young adults [16], which found no significant difference in depression between one- and multiple-person households. However, because that study defined depressive symptoms using a higher PHQ-9 cutoff (≥10) and included different covariates, the two results cannot be directly compared. One possible explanation for the association observed in our study is that social restrictions during the pandemic may have intensified the loneliness and social isolation experienced by young adults living alone; however, this interpretation should be tested in future studies that compare the same measures across periods.

In our study, education and marital status were significantly associated with depressive symptoms in men but not in women. In a systematic review of factors associated with depression [17], education was significant in only a few of the 29 studies included, whereas marital status was significant in 26 of the 29 studies, indicating some inconsistency across studies. The absence of these associations in women may be related to sociocultural factors that differ between men and women; however, these factors could not be examined in this study, and further research is needed to clarify these sex differences.

In our study, age and area of residence were not significantly associated with depressive symptoms in either men or women, whereas occupation was significantly associated with depressive symptoms only in men. In terms of age, a study using the pre-pandemic 2017 Community Health Survey [15] found no significant difference in the risk of depression between men aged 19–29 years and those aged 30–39 years but reported that the risk of depression was significantly lower in women aged 30–39 years compared to those aged 19–29 years; this differs from our findings. On the other hand, a 2020 study conducted during the COVID-19 pandemic [33] found no differences in the risk of depressive symptoms by age, which was consistent with our findings. However, the study did not analyze its results by sex; accordingly, it did not examine sex differences.

Regarding occupation, an earlier systematic review [17] reported a consistent association between unemployment and depression. In our study, not having an occupation was associated with higher odds of depressive symptoms only in men, which is partly consistent with this review. In contrast, household income was significantly associated with depressive symptoms in both men and women, suggesting that economic stability at the household level, which may reflect financial burden that is difficult to capture with objective measures, is an important correlate of depressive symptoms regardless of sex [18].

Area of residence was included in our study based on a literature review [18] reporting that depression is associated with region and the built environment; however, area of residence was not significantly associated with depressive symptoms in either men or women. This discrepancy may be attributable to differences in variable definitions. The earlier studies [18] mostly measured the environment as subjectively experienced by participants, such as social disorder or neighborhood cohesion, whereas we measured only the objective environment, such as population size or degree of urbanization. Therefore, depressive symptoms may be more closely related to individuals’ subjective perceptions of their environment than to the objective characteristics of their area of residence.

Among the health-related characteristics, smoking status and sleep duration were significantly associated with depressive symptoms in both men and women. This result is consistent with an earlier study [15], in which smoking was associated with 1.17- and 1.99-fold higher odds of depression in men and women, respectively, and with another study [16] reporting that smokers had 1.92 times higher odds of depression than nonsmokers. Thus, smoking was consistently associated with depressive symptoms in both sexes, with a stronger association in women.

Compared with normal sleep duration (7–9 h), both short (<7 h) and long (>9 h) sleep durations were associated with higher odds of depressive symptoms in both sexes. In a meta-analysis of sleep duration and mental disorders in adults [19], the pooled results of cohort studies showed that short sleep duration was associated with a 1.43-fold higher risk of depression, whereas long sleep duration was not significantly associated with depression. Accordingly, insufficient sleep appears to be consistently associated with depressive symptoms, whereas further research is needed to determine whether excessive sleep is associated with depressive symptoms in young adults.

In our study, obesity based on BMI was associated with lower odds of depressive symptoms in both men and women, although the relevant BMI category differed by sex (obesity in men and overweight in women). This is consistent with an earlier study [15] that reported an association between overweight or obesity and a lower risk of depression compared with non-obesity in both men and women. The difference in the relevant BMI category between men and women in our study may be related to differences in BMI distribution, as only 17.5% of women were obese, compared with 45.0% of men. In addition, the earlier study included adults aged ≥ 19 years, whereas our study was limited to young adults aged 19–39 years, and the proportions of nonobese participants differed between the studies (55.0% of men and 82.5% of women in our study vs. 38.4% of men and 57.5% of women in the earlier study). Furthermore, because BMI and body shape perception were entered simultaneously in the models, the inverse association between BMI and depressive symptoms may partly reflect adjustment for body shape perception; that is, among individuals with the same perceived body shape, those with a higher BMI may have had lower odds of depressive symptoms. Therefore, the association between BMI and depressive symptoms in young adults should be interpreted together with body shape perception, as discussed below.

Binge drinking frequency was significantly associated with depressive symptoms only in women, and the odds of depressive symptoms were higher with greater binge drinking frequency. This is consistent with an earlier study [15], in which high-risk drinking was associated with 1.43-fold higher odds of depression in women but not in men. Furthermore, a systematic review [17] reported that the relationship between drinking and depression is ambiguous and that low or moderate alcohol consumption, which is fundamentally different from alcohol abuse, may be protective against depression owing to its relationship with social adaptation. Because men may obtain more social benefits from drinking than women [17], this sex difference requires further investigation.

Physical activity was not significantly associated with depressive symptoms in either men or women in our study. In an earlier study [15], non-walkers had 1.32 (men) and 1.25 (women) times higher odds of depression than regular walkers, whereas another study [16] reported no significant association between walking and depression. These inconsistent findings suggest the need for further research on the association between physical activity and depressive symptoms in young adults.

All the psychological characteristics analyzed in our study—subjective health status, subjective stress, and subjective body shape perception—were significantly associated with depressive symptoms in both men and women. In particular, poor subjective health status showed one of the strongest associations with depressive symptoms. This finding is similar to that of an earlier study [15], in which poor perceived health status was associated with 4.16- and 2.75-fold higher odds of depression in men and women, respectively, suggesting a close relationship between perceived physical health and mental health.

High subjective stress also showed one of the strongest associations with depressive symptoms in both sexes. These results are consistent with earlier research [15], in which high perceived stress was associated with 9.29- and 8.39-fold higher odds of depression in men and women, respectively. In addition, 30.4% of the participants in a Korean study conducted during the COVID-19 pandemic [33] reported increased stress at home, which appeared to be associated with depression. Although our cross-sectional data cannot determine whether stress levels changed during the pandemic, these findings suggest that daily stress is an important target in depression screening among young adults, and longitudinal studies are needed to examine whether pandemic-related stress contributed to depressive symptoms.

Regarding subjective body shape perception, perceiving oneself as either skinny or obese was associated with higher odds of depressive symptoms in men, whereas only perceiving oneself as obese was associated with depressive symptoms in women. The proportion of participants classified as obese based on BMI was 45.0% in men and 17.5% in women, whereas the proportions of those who perceived their own body shape as obese were 45.8% and 39.2%, respectively. Thus, men’s perceptions of their body shape were relatively consistent with their BMI, whereas women were more likely to perceive themselves as obese than their BMI indicated. A systematic review [20] reported that women tend to overestimate their body size or be dissatisfied with their body image and that such negative body image perceptions are associated with depression irrespective of BMI. Similarly, in our study, obesity based on BMI was not associated with higher odds of depressive symptoms in either sex, whereas perceiving oneself as obese was associated with higher odds in both sexes, suggesting that body image perception may be a more relevant consideration than actual body weight when screening for depressive symptoms in young adults.

These findings have several public health implications for depression screening, prevention, and mental health support for young adults in Korea. First, high subjective stress, poor subjective health status, and short or long sleep duration were strongly associated with depressive symptoms in both sexes; thus, brief assessments of perceived stress, self-rated health, and sleep may help identify young adults who require depression screening in community health settings. Second, low household income and living alone were associated with depressive symptoms in both sexes, suggesting that young adults with economic difficulties or those living alone should be prioritized in mental health outreach and linked to mental health services. Third, sex-specific approaches may be needed. For young men, who are less likely than young women to use mental health services [14], proactive outreach may be particularly important for those with lower education, those who are separated, widowed, or divorced, those without an occupation, and those who perceive themselves as skinny. For young women, programs addressing smoking and binge drinking, together with interventions addressing negative body image perception, may be useful components of depression prevention. Finally, because psychosocial interventions can reduce depressive symptoms [11] and many young adults seek help only after their symptoms worsen [13], integrating routine depression screening that considers these characteristics into existing health services for young adults may facilitate earlier detection and treatment, particularly during public health crises that restrict social activities.

This study used data from a large-scale, nationally representative survey in South Korea to identify common and sex-specific factors associated with depressive symptoms among young adults, thereby providing basic data for future depressive symptom screening in this population. Nevertheless, the findings should be interpreted with caution because of the following limitations. First, because this was a cross-sectional study, the temporal relationships between depressive symptoms and the associated factors could not be determined, and the identified factors cannot be interpreted as causes of depressive symptoms. In addition, because the data were collected at a single time point in 2022 without a pre-pandemic comparison using the same measures, it was not possible to determine whether the observed associations changed as a result of the COVID-19 pandemic. Therefore, the pandemic should be regarded as the context in which the data were collected, and the pandemic-related interpretations in this study should be considered hypotheses to be tested in future longitudinal or repeated cross-sectional studies. Second, depressive symptoms were assessed using the self-reported PHQ-9 with a cutoff of 6 points, which prioritizes sensitivity; thus, the outcome represents a positive screening result rather than a clinical diagnosis of depressive disorder, and the proportion of participants with depressive symptoms may have been overestimated. Third, sex differences were identified by comparing the sex-stratified models, and formal tests of interaction between sex and each factor were not performed; therefore, differences in statistical significance between men and women should be interpreted with caution. Finally, because this was a secondary data analysis, only the variables available in the original survey could be analyzed; therefore, other potentially relevant factors identified in earlier studies could not be considered, and some variable definitions differed from those used in earlier research.

## 5. Conclusions

This study used data from the 2022 Community Health Survey to identify the factors associated with depressive symptoms among Korean young adults by sex in the third year of the COVID-19 pandemic. Women had higher odds of depressive symptoms than men. High subjective stress, poor subjective health status, short or long sleep duration, perceiving oneself as obese, living alone, low household income, and smoking were associated with depressive symptoms in both sexes, whereas lower education; being separated, widowed, or divorced; having no occupation; and perceiving oneself as skinny were associated with depressive symptoms only in men, and binge drinking was associated with depressive symptoms only in women. These findings suggest that sex differences should be considered when screening for depressive symptoms among young adults and that young adults with low household income or those living alone may require particular attention. Further research should develop and evaluate depressive symptom screening systems and intervention programs that account for sex differences in young adults.

## Figures and Tables

**Table 1:** Participants’ General, Health-Related, and Psychological Characteristics and Depressive Symptoms.

Characteristics	Categories	Total (*N* = 47,353)	Men (*n* = 23,407)	Women (*n* = 23,946)	Rao–Scott χ^2^	*p*
		*n* (%) ^†^	*n* (%) ^†^	*n* (%) ^†^		
Age (year)	19–29	22,667 (51.3)	11,229 (51.6)	11,438 (51.0)	1.56	0.212
	30–39	24,686 (48.7)	12,178 (48.4)	12,508 (49.0)		
Education level	<University	26,770 (55.8)	13,933 (59.1)	12,837 (52.3)	193.25	<0.001
	≥University	20,583 (44.2)	9474 (40.9)	11,109 (47.7)		
Marital status	Single	29,654 (65.2)	16,178 (70.8)	13,476 (59.2)	377.69	<0.001
	Married	16,640 (33.0)	6873 (28.0)	9767 (38.3)		
	SWD	1059 (1.8)	356 (1.2)	703 (2.4)		
Occupation	Yes	33,166 (69.3)	17,854 (74.6)	15,312 (63.5)	490.69	<0.001
	No	14,187 (30.7)	5553 (25.4)	8634 (36.5)		
Household income (10,000 won)	<500	24,122 (48.7)	12,311 (50.2)	11,811 (47.1)	44.99	<0.001
	≥500	23,231 (51.3)	11,096 (49.8)	12,135 (52.9)		
Number of household members	1	7375 (15.8)	4506 (18.8)	2869 (12.5)	282.07	<0.001
	≥2	39,978 (84.2)	18,901 (81.2)	21,077 (87.5)		
Residential area	Metropolitan	17,222 (45.9)	8370 (44.8)	8852 (47.3)	21.66	<0.001
	Middle & small	18,431 (41.4)	9150 (41.8)	9281 (40.9)		
	Rural	11,700 (12.7)	5887 (13.4)	5813 (11.8)		
Smoking	No	36,522 (76.3)	14,234 (61.4)	22,288 (92.5)	5128.71	<0.001
	Yes	10,831 (23.7)	9173 (38.6)	1658 (7.5)		
Frequency of binge drinking	None	18,721 (38.3)	7428 (31.5)	11,293 (45.8)	450.08	<0.001
	≤1/week	27,050 (58.4)	14,929 (64.2)	12,121 (52.0)		
	Almost every day	1582 (3.3)	1050 (4.3)	532 (2.2)		
Physical activity	Inactive	14,583 (28.7)	5712 (23.1)	8871 (34.8)	798.47	<0.001
	Minimal	22,002 (48.4)	10,403 (46.1)	11,599 (50.9)		
	Healthy	10,768 (22.9)	7292 (30.8)	3476 (14.3)		
Obesity	Underweight	3296 (7.2)	467 (2.1)	2829 (12.7)	1843.38	<0.001
	Normal	19,911 (42.2)	6639 (29.3)	13,272 (56.3)		
	Overweight	8872 (18.8)	5511 (23.6)	3361 (13.5)		
	Obese	15,274 (31.8)	10,790 (45.0)	4484 (17.5)		
Sleep duration (hour/day)	<7	17,498 (38.0)	9242 (40.3)	8256 (35.4)	65.01	<0.001
	7–9	28,345 (58.8)	13,554 (57.1)	14,791 (60.8)		
	>9	1510 (3.2)	611 (2.6)	899 (3.8)		
Subjective health status	Good	29,213 (62.2)	15,205 (65.1)	14,008 (59.1)	74.10	<0.001
	Average	15,932 (33.3)	7181 (30.6)	8751 (36.1)		
	Bad	2208 (4.5)	1021 (4.3)	1187 (4.8)		
Subjective stress	Low	34,750 (73.2)	17,765 (75.6)	16,985 (70.6)	117.80	<0.001
	High	12,603 (26.8)	5642 (24.4)	6961 (29.4)		
Subjective body shape perception	Skinny	6948 (15.4)	3657 (16.1)	3291 (14.5)	128.37	<0.001
	Normal	19,931 (42.0)	8962 (38.1)	10,969 (46.3)		
	Obese	20,474 (42.6)	10,788 (45.8)	9686 (39.2)		
Depressive symptoms	No	41,380 (87.0)	21,156 (89.9)	20,224 (83.7)	312.06	<0.001
	Yes	5973 (13.0)	2251 (10.1)	3722 (16.3)		

SWD = separation, widowed, divorce. ^†^ Data are unweighted number and weighted proportions.

**Table 2:** Differences in Depressive Symptoms by General, Health-related, and Psychological Characteristics of Participants.

Characteristics	Categories		Total (*N* = 47,353)		Men (*n* = 23,407)			Women (*n* = 23,946)		
		None DS	DS	Rao–Scott	None DS	DS	Rao–Scott χ^2^ *(p)*	None DS	DS	Rao–Scott χ^2^ *(p)*
		(*n* = 41,380)	(*n* = 5973)	χ^2^ *(p)*	(*n* = 21,156)	(*n* = 2251)		(*n* = 20,224)	(*n* = 3722)	
		*n* (%) ^†^	*n* (%) ^†^		*n* (%) ^†^	*n* (%) ^†^		*n* (%) ^†^	*n* (%) ^†^	
Sex	Men	21,156 (53.9)	2251 (40.3)	312.06	-	-	-	-	-	-
	Women	20,224 (46.1)	3722 (59.7)	(<0.001)	-	-	-	-	-	-
Age (year)	19–29	19,928 (51.7)	2739 (49.0)	11.72	10,228 (52.0)	1001 (48.6)	7.9	9700 (51.3)	1738 (49.4)	4.11
	30–39	21,452 (48.3)	3234 (51.0)	−0.001	10,928 (48.0)	1250 (51.4)	−0.005	10,524 (48.7)	1984 (50.6)	−0.043
Education level	<University	23,157 (55.2)	3613 (59.7)	30.83	12,480 (58.4)	1453 (65.2)	33.23	10,677 (51.6)	2160 (55.9)	19.07
	≥University	18,223 (44.8)	2360 (40.3)	(<0.001)	8676 (41.6)	798 (34.8)	(<0.001)	9547 (48.4)	1562 (44.1)	(<0.001)
Marital status	Single	25,934 (65.4)	3720 (64.4)	23.57	14,617 (70.8)	1561 (70.4)	16.53	11,317 (58.9)	2159 (60.5)	9.08
	Married	14,584 (33.0)	2056 (32.6)	(<0.001)	6247 (28.1)	626 (27.1)	(<0.001)	8337 (38.8)	1430 (36.2)	(<0.001)
	SWD	862 (1.6)	197 (3.0)		292 (1.1)	64 (2.5)		570 (2.3)	133 (3.3)	
Occupation	Yes	28,983 (69.3)	4183 (69.4)	0.02	16,120 (74.5)	1734 (75.7)	1.4	12,863 (63.2)	2449 (65.1)	4.02
	No	12,397 (30.7)	1790 (30.6)	−0.881	5036 (25.5)	517 (24.3)	−0.237	7361 (36.8)	1273 (34.9)	−0.045
Household income (10,000 won)	<500	20,613 (47.5)	3509 (57.1)	143.45	10,964 (49.4)	1347 (57.8)	47.28	9649 (45.2)	2162 (56.6)	128.5
	≥500	20,767 (52.5)	2464 (42.9)	(<0.001)	10,192 (50.6)	904 (42.2)	(<0.001)	10,575 (54.8)	1560 (43.4)	(<0.001)
Number of household members	1	6126 (15.1)	1249 (20.6)	101.66	3922 (18.2)	584 (23.8)	39.63	2204 (11.4)	665 (18.4)	134.21
	≥2	35,254 (84.9)	4724 (79.4)	(<0.001)	17,234 (81.8)	1667 (76.2)	(<0.001)	18,020 (88.6)	3057 (81.6)	(<0.001)
Residential area	Metropolitan	14,962 (45.9)	2260 (46.6)	2.21	7522 (44.7)	848 (45.8)	0.52	7440 (47.3)	1412 (47.1)	2.78
	Middle & small	16,019 (41.3)	2412 (41.6)	−0.112	8272 (41.9)	878 (41.0)	−0.589	7747 (40.7)	1534 (42.1)	−0.065
	Rural	10,399 (12.8)	1301 (11.8)		5362 (13.4)	525 (13.2)		5037 (12.0)	776 (10.8)	
Smoking	No	32,270 (77.2)	4252 (70.0)	123.1	13,100 (62.7)	1134 (50.1)	120.28	19,170 (94.2)	3118 (83.4)	429.05
	Yes	9110 (22.8)	1721 (30.0)	(<0.001)	8056 (37.3)	1117 (49.9)	(<0.001)	1054 (5.8)	604 (16.6)	(<0.001)
Frequency of binge drinking	None	16,561 (38.7)	2160 (35.9)	32.95	6733 (31.6)	695 (30.0)	15.81	9828 (47.0)	1465 (39.9)	49.26
	≤1/week	23,556 (58.3)	3494 (59.0)	(<0.001)	13,534 (64.3)	1395 (63.2)	(<0.001)	10,022 (51.2)	2099 (56.1)	(<0.001)
	Almost every day	1263 (3.0)	319 (5.1)		889 (4.1)	161 (6.8)		374 (1.8)	158 (4.0)	
Physical activity	Inactive	12,557 (28.2)	2026 (32.0)	15.21	5087 (22.8)	625 (26.5)	10.83	7470 (34.6)	1401 (35.7)	5.38
	Minimal	19,404 (48.8)	2598 (45.6)	(<0.001)	9520 (46.6)	883 (41.4)	(<0.001)	9884 (51.4)	1715 (48.5)	−0.005
	Healthy	9419 (23.0)	1349 (22.4)		6549 (30.6)	743 (32.1)		2870 (14.0)	606 (15.8)	
Obesity	Underweight	2785 (7.0)	511 (8.5)	12.23	399 (2.0)	68 (3.3)	9.91	2386 (12.8)	443 (12.0)	41.82
	Normal	17,541 (42.6)	2370 (40.2)	(<0.001)	6070 (29.7)	569 (26.2)	(<0.001)	11,471 (57.6)	1801 (49.7)	(<0.001)
	Overweight	7887 (19.0)	985 (17.1)		5044 (23.9)	467 (21.7)		2843 (13.4)	518 (14.0)	
	Obese	13,167 (31.4)	2107 (34.2)		9643 (44.4)	1147 (48.8)		3524 (16.2)	960 (24.3)	
Sleep duration (hour/day)	<7	14,558 (36.3)	2940 (49.4)	210	8097 (39.2)	1145 (51.0)	74.91	6461 (32.9)	1795 (48.5)	162.79
	7–9	25,597 (60.8)	2748 (45.8)	(<0.001)	12,546 (58.4)	1008 (44.8)	(<0.001)	13,051 (63.6)	1740 (46.4)	(<0.001)
	>9	1225 (2.9)	285 (4.8)		513 (2.4)	98 (4.2)		712 (3.5)	187 (5.1)	
Subjective health status	Good	27,272 (66.7)	1941 (32.4)	1537.89	14,429 (68.6)	776 (33.8)	640.28	12,843 (64.5)	1165 (31.5)	938.8
	Average	12,893 (30.5)	3039 (51.6)	(<0.001)	6078 (28.4)	1103 (50.3)	(<0.001)	6815 (33.0)	1936 (52.4)	(<0.001)
	Bad	1215 (2.8)	993 (16.0)		649 (3.0)	372 (15.9)		566 (2.5)	621 (16.1)	
Subjective stress	Low	32,806 (79.2)	1944 (33.2)	4446.23	16,993 (80.3)	772 (33.9)	1990.24	15,813 (77.9)	1172 (32.6)	2503.55
	High	8574 (20.8)	4029 (66.8)	(<0.001)	4163 (19.7)	1479 (66.1)	(<0.001)	4411 (22.1)	2550 (67.4)	(<0.001)
Subjective body shape perception	Skinny	6061 (15.3)	887 (15.7)	164.54	3251 (15.8)	406 (19.3)	77.79	2810 (14.7)	481 (13.3)	135.07
	Normal	18,093 (43.7)	1838 (30.6)	(<0.001)	8391 (39.6)	571 (24.9)	(<0.001)	9702 (48.6)	1267 (34.5)	(<0.001)
	Obese	17,226 (41.0)	3248 (53.7)		9514 (44.6)	1274 (55.8)		7712 (36.7)	1974 (52.2)	

DS = depressive symptoms; SWD = separation, widowed, divorce. ^†^ Data are unweighted number and weighted proportions.

**Table 3:** Factors Associated with Depressive Symptoms of Participants.

Characteristics	Categories	Total (*N* = 47,353)		Men (*n* = 23,407)		Women (*n* = 23,946)	
		Adjusted OR (95% CI)	*p*	Adjusted OR (95% CI)	*p*	Adjusted OR (95% CI)	*p*
Sex	Men	1					
	Women	1.89 (1.72–2.07)	<0.001				
Age (year)	19–29	1.05 (0.96–1.14)	0.274	1.05 (0.92–1.19)	0.486	1.00 (0.90–1.12)	0.969
	30–39	1		1		1	
Education level	<University	1.10 (1.02–1.19)	0.012	1.27 (1.13–1.42)	<0.001	0.98 (0.89–1.08)	0.655
	≥University	1		1		1	
Marital status	Single	1.13 (1.03–1.24)	0.011	1.09 (0.93–1.26)	0.284	1.12 (0.99–1.25)	0.066
	Married	1		1		1	
	SWD	1.44 (1.16–1.78)	0.001	2.42 (1.71–3.42)	<0.001	1.10 (0.85–1.42)	0.466
Occupation	No	1.08 (1.00–1.17)	0.051	1.16 (1.01–1.33)	0.034	1.04 (0.94–1.14)	0.475
	Yes	1		1		1	
Household income (10,000 won)	<500	1.22 (1.13–1.32)	<0.001	1.17 (1.04–1.31)	0.008	1.24 (1.13–1.37)	<0.001
	≥500	1		1		1	
Number of household members	1	1.25 (1.14–1.38)	<0.001	1.23 (1.07–1.41)	0.004	1.27 (1.11–1.46)	<0.001
	≥2	1		1		1	
Smoking	No	1		1		1	
	Yes	1.39 (1.27–1.52)	<0.001	1.16 (1.04–1.29)	0.006	1.92 (1.67–2.22)	<0.001
Frequency of binge drinking	None	1		1		1	
	≤1/week	1.13 (1.05–1.21)	0.001	1.05 (0.93–1.17)	0.440	1.19 (1.08–1.31)	<0.001
	Almost every day	1.41 (1.18–1.67)	<0.001	1.24 (0.99–1.57)	0.065	1.60 (1.23–2.08)	<0.001
Physical activity	Inactive	1		1		1	
	Minimal	0.99 (0.91–1.07)	0.709	0.96 (0.84–1.10)	0.547	1.00 (0.91–1.10)	0.960
	Healthy	1.12 (1.02–1.24)	0.020	1.14 (0.99–1.31)	0.077	1.10 (0.96–1.26)	0.176
Obesity	Underweight	1.06 (0.91–1.23)	0.462	1.22 (0.87–1.73)	0.247	1.13 (0.95–1.34)	0.176
	Normal	1		1		1	
	Overweight	0.95 (0.85–1.06)	0.344	1.09 (0.91–1.30)	0.334	0.82 (0.70–0.97)	0.016
	Obese	0.80 (0.71–0.90)	<0.001	0.76 (0.62–0.92)	0.006	0.90 (0.78–1.03)	0.117
Sleep duration (hour/day)	<7	1.49 (1.39–1.60)	<0.001	1.39 (1.25–1.55)	<0.001	1.57 (1.43–1.72)	<0.001
	7–9	1		1		1	
	>9	1.91 (1.60–2.27)	<0.001	1.93 (1.42–2.63)	<0.001	1.89 (1.53–2.33)	<0.001
Subjective health status	Good	1		1		1	
	Average	2.47 (2.29–2.67)	<0.001	2.68 (2.39–3.01)	<0.001	2.33 (2.12–2.56)	<0.001
	Bad	6.20 (5.49–7.00)	<0.001	5.65 (4.72–6.78)	<0.001	6.61 (5.62–7.77)	<0.001
Subjective stress	Low	1		1		1	
	High	5.61 (5.23–6.02)	<0.001	6.16 (5.52–6.88)	<0.001	5.28 (4.82–5.79)	<0.001
Subjective body shape perception	Skinny	1.27 (1.12–1.43)	<0.001	1.43 (1.19–1.72)	<0.001	1.09 (0.92–1.29)	0.313
	Normal	1		1		1	
	Obese	1.61 (1.46–1.77)	<0.001	1.63 (1.39–1.91)	<0.001	1.65 (1.46–1.87)	<0.001

CI = confidence interval; OR = odds ratio; SWD = separation, widowed, divorce. The pooled model for the total sample was additionally adjusted for sex; all models included all variables shown in the table.

## Data Availability

The raw data used in this study were provided by the Korean Community Health Survey and can be downloaded from the agency’s website (https://chs.kdca.go.kr/chs/; accessed on 29 June 2024) through a simple procedure.

## Supplementary Materials

The following supporting information can be downloaded at https://www.mdpi.com/article/10.3390/healthcare14193356/s1: Table S1: Multiple logistic regression results for depressive symptoms among Korean young adults in an earlier study [16].
