# Supplementary material for: Factors Associated with Depressive Symptoms Among Korean Young Adults by Sex in the Third Year of the COVID-19 Pandemic
Source: Healthcare (Basel). 2026 Oct 8;14(19):3356. doi: 10.3390/healthcare14193356 (PMC13664773; doi:10.3390/healthcare14193356)
Supplement: Supplementary file 1 [file healthcare-14-03356-s001.zip › healthcare-4583480-supplementary.pdf]

Supplementary Materials

Factors associated with depressive symptoms among Korean young adults by sex in the third year of the COVID-19 pandemic

Table S1. Multiple logistic regression results for depressive symptoms among Korean young adults in an earlier study (Reference 16).

Characteristics	Categories	OR	*p*
Sex	Men	1	
	Women	2.62	<.001
Marital status	Married	1	
	Single (no spouse)	1.92	<.001
Education level	University graduate	1	
	High school graduate	1.15	.045
Living type	Multi-person household	1	
	One-person household	1.02	.816
Household income (10,000 won)	>400	1	
	200–400	1.09	.231
	100–200	1.47	<.001
	<100	1.71	<.001
Basic livelihood security recipient	No	1	
	Yes	1.42	.025
	In the past	1.34	.298
Food security	Secured	1	
	Not secured	1.92	<.001
Current smoking	No	1	
	Yes	1.92	<.001
High-risk drinking	No	1	
	Yes	1.52	<.001
Walking	Yes	1	
	No	0.91	.113
Positive attitude toward the local social and physical environment	6–7 points	1	
	3–5 points	2.06	<.001
	1–2 points	3.65	<.001
	0 points	6.10	<.001
Social network	3 points	1	
	2 points	1.04	.747
	1 point	1.12	.304
	0 points	1.46	.002
Religious activities	Yes	1	
	No	0.90	.202
Social activities	Yes	1	
	No	1.72	<.001
Leisure activities	Yes	1	
	No	1.00	.960

Characteristics	Categories	OR	*p*
Charity activities	Yes	1	
	No	0.90	.482

OR = odds ratio. Data are from the 2017 Community Health Survey (adults aged 19–39 years, N = 47,239). Depressive symptoms were defined as a Patient Health Questionnaire-9 score ≥10, and all variables were entered simultaneously into a complex sample logistic regression model. Odds ratios and p-values are presented as reported in Table 4 of the original article.

Source: Oh, C.H.; Yu, B.C.; Lee, M.J.; Kang, S.H. Factors related to the prevalence of depression among young people. Korean J. Health Serv. Manag. 2021, 15, 27–39. https://doi.org/10.12811/kshsm.2021.15.2.027
